# Body image, visual working memory and visual mental imagery

**DOI:** 10.7717/peerj.775

**Published:** 2015-02-17

**Authors:** Stephen Darling, Clare Uytman, Richard J. Allen, Jelena Havelka, David G. Pearson

**Affiliations:** 1 Division of Psychology and Sociology, Queen Margaret University, Edinburgh, UK; 2 School of Psychology, University of Leeds, UK; 3 School of Psychology, University of Aberdeen, UK

**Keywords:** Body dissatisfaction, Body image, Visual imagery, Visual memory, Working memory

## Abstract

Body dissatisfaction (BD) is a highly prevalent feature amongst females in society, with the majority of individuals regarding themselves to be overweight compared to their personal ideal, and very few self-describing as underweight. To date, explanations of this dramatic pattern have centred on extrinsic social and media factors, or intrinsic factors connected to individuals’ knowledge and belief structures regarding eating and body shape, with little research examining links between BD and basic cognitive mechanisms. This paper reports a correlational study in which visual and executive cognitive processes that could potentially impact on BD were assessed. Visual memory span and self-rated visual imagery were found to be predictive of BD, alongside a measure of inhibition derived from the Stroop task. In contrast, spatial memory and global precedence were not related to BD. Results are interpreted with reference to the influential multi-component model of working memory.

## Body Image, Visual Working Memory and Visual Mental Imagery

Body image is a general term attached to a complex web of constructs by which individuals relate to their own bodies, and in particular to their appearance. In western societies the issue of weight (thinness) has been of particular significance for body image, as societal ideals have tended to emphasize unattainable degrees of thinness, especially in females (Tiggemann, 2006). These unrealistic ideals may underlie what has been termed a ‘normative discontent’ (Rodin, Silberstein & Striegel-Moore, 1984). There is little doubt that body dissatisfaction (BD) contributes greatly to levels of distress in the general population (Ohring, Graber & Brooks-Gunn, 2002; Thompson, 1999), and is related to onset of pathological eating disorders (Attie & Brooks-Gunn, 1989; Killen et al., 1996). Furthermore, although females and males both show evidence of BD at roughly equivalent prevalence (e.g., Furnham & Calnan, 1998; Furnham, Badmin & Sneade, 2002), the nature of dissatisfaction is qualitatively different between the sexes; the vast majority of females expressing BD express a preference for a slimmer figure, whilst for males the distribution is roughly symmetrical, with many wishing to be bulkier (Furnham & Calnan, 1998; Furnham, Badmin & Sneade, 2002; Drewnowski & Yee, 1987).

An increased knowledge of the predictors and correlates of BD is necessary in order to further understand this issue. A good deal is already understood about such predictors; factors such as environmental social pressure to be thin, weight-related teasing, internalization of a thin-ideal, lack of social support, individual differences in body mass and peer pressure have all been identified as specific predictors of body dissatisfaction in adolescent girls (Stice & Whitenton, 2002; Presnell, Bearman & Stice, 2004).

Although extrinsic social forces such as those detailed above undoubtedly shape body dissatisfaction, it is important to probe intrinsic factors that may be related to negative body image evaluation too. There is, for example, clear evidence for the existence of a relationship between negative affect and body dissatisfaction (Presnell, Bearman & Stice, 2004; Kostanski & Gullone, 1998), though longitudinal research suggests that early BD predicts later depression rather than vice versa (Stice et al., 2000; Paxton et al., 2006). Other intrinsic factors linked to BD include dispositional factors, personality factors and attachment (Abbate-Daga et al., 2010), alongside biological factors such as adiposity (e.g., Stice & Whitenton, 2002).

Information-processing factors also have the potential to impact on BD. In the last twenty years an understanding of the constraints imposed by cognitive architecture has had a profound impact on understanding of social processing (see, e.g., Fazio et al., 1995; Greenwald, Nosek & Banaji, 2003). In a similar vein, a body of research has emerged focusing on information processing and BD (for a review see Lee & Shafran, 2004). This research has broadly shown that individuals with eating disorders attend differentially to information pertaining to shape, weight and eating (e.g., Vitousek & Hollon, 1990) and body components (Freeman et al., 1991; Fairburn, Shafran & Cooper, 1999). There is also a range of evidence from modifications of the Stroop task (Stroop, 1935) where participants are required to report the colour of ink in which a word is printed. In the emotional variant of the Stroop task, neutral and emotionally charged words are presented to participants who are then asked to identify the print colour of those words. It has been demonstrated that unpleasant words (e.g., fear) produce greater slowing down of reaction times than neutral words (e.g., leaf). This pattern has also been observed in clinical populations. For example, words related to specific phobias (i.e., arachnophobia) result in slower response times when presented to individuals with those phobias (for a review see Williams, Mathews & MacLeod, 1996), while alcohol related words produce slower response times in alcoholics (e.g., Lusher, Chandler & Ball, 2004). In relation to the present study, participants with a high ‘drive for thinness’ demonstrated greater impairment in ink colour naming latency when the ostensibly irrelevant text was composed of food words than control words (Perpiñá et al., 1993); participants who were high in dietary restraint showed a similar pattern towards food and body shape words (Green & Rogers, 1993). There is also a relationship between the retention of body-related items in memory and BD (Baker, Williamson & Sylve, 1995). Hargreaves & Tiggemann (2002) reported that declines in girls’ body satisfaction were related to the strength of cognitive schemata underlying appearance at the start of the research period, and that this was distinguishable from self-esteem. The picture emerging from this literature is that of a relationship between biases in cognition and information processing centred around concepts related to the body and food, and increased body dissatisfaction or proneness to eating disorders.

The cognitive factors described above, however, are intrinsically linked to the conceptual understanding of bodies and food, and are likely to reflect the content of semantic memory related to food and body-related self-image. There are a further set of cognitive functions that may influence body dissatisfaction, these being more abstract cognitive processes that likely influence a range of behaviours such as, for example, planning and inhibition. There is, for example, evidence that poor inhibition, as assessed on the colour-word Stroop task, is associated with increased obesity, though not with anorexia nervosa (Fagundo et al., 2012), alongside other evidence of links between obesity and impaired executive functions such as inhibition, flexibility and planning (Boeka & Lokken, 2008; Cserjési et al., 2009; Cserjési et al., 2007; Gunstad et al., 2007; Nederkoorn et al., 2006a; Nederkoorn et al., 2006b). There are also relationships between eating disorders and executive functions (for reviews see: Aspen, Darcy & Lock, 2013; Dobson & Dozois, 2004; Lena, Fiocco & Leyenaar, 2004; Roberts et al., 2007).

Our purpose here is to focus in more detail on one particular aspect of cognition that has been largely neglected in understanding body dissatisfaction and related issues: visual cognition. The research reported here starts from a simple and relatively uncontroversial position—that there are (at least) two representative codes capable of sustaining imagery in the cognitive system, one verbal and one visuospatial in nature (Paivio, 1991). This dual coding approach underlies the highly influential Working Memory approach (Baddeley & Hitch, 1974; Baddeley, Allen & Hitch, 2011). In essence the working memory approach posits that parallel temporary verbal and visual information storage systems (known respectively as the phonological loop and visuo-spatial sketch pad) store information in a passive manner, whilst other multimodal systems can perform operations on or form links to that information. One type of multimodal system is the ‘central executive’—a label reflecting a set of largely active processes that can manipulate the information held temporarily active in the passive stores, whilst the another, termed the ‘episodic buffer’ is more automatic in nature and involved in the formation of cohesive and unified ‘episodes’ (Baddeley, Allen & Hitch, 2011). When looked at from this perspective it seems likely that any representation of body image is likely to contain a visuospatial component alongside a verbal one. There is ample evidence that such visual images can be created and maintained in memory, even in the absence of explicit visual input (e.g., Quinn & McConnell, 1996; Pearson & Logie, 2004). Any task where a participant is asked to consider their own body image should invoke, at least to some extent, a visual representation. Hence, visual imagery ability should be linked to body image. One possibility is that the more realistic and veridical a participant’s internal model of their own appearance is, the more resistant they should be to bias and distortion of body image driven by social and cultural factors operationalized in semantic memory.

Smeets et al. (2009) have postulated a link between visual imagery processes and body image using a variant of the Distance Comparison task (Kosslyn, Ball & Reiser, 1978). In their study participants were asked to mentally scan across two specified body widths (e.g., hips and waist) and indicate as quickly as possible which body part was longer or shorter. The results showed females with high BD were significantly less accurate on body width comparisons involving small (less than 9 cm) differences, implying that they had a less accurate visual image of their own body in comparison to a group of low BD females.

However, the Distance Comparison task used by Smeets et al. is a behavioural measure that cannot directly assess a participant’s subjective experience of mental imagery. Auchus, Kose & Allen (1993) report a study that, surprisingly, appears to be the only attempt to date to study the relationship between subjective self-reported visual imagery and body imagery. This study subdivided a sample on a post-hoc basis into four groups based on a test where they had to adjust the appearance of an initially distorted image of themselves until they felt it reflected reality. In a control test they adjusted the shape of an object (a tin of soup) in the same way. Participants were assessed to be either non-distorters, distorters of object but not own-body representations, distorters of own-body representations but not objects, and general distorters. Various different measures of visual imagery were taken: overall, no significant group differences were identified. The authors also adopted an extreme groups approach and claimed to observe increased ratings of imagery vividness (using the Vividness of Visual Imagery Questionnaire—VVIQ: Marks, 1973) when a subset of the participants who distorted body image the most was compared against the subset that distorted the least. Such an approach is methodologically problematic when coupled with small sample size, and does not appear to have been replicated in the literature. In order to clarify and consolidate understanding of the relationship of visual imagery and body imagery, the current study adopts a correlational design in which a number of cognitive measures are adopted in order to predict BD in a female sample. We focus specifically on females because the aforementioned evidence that BD has a quite different profile in females and males would imply that separate consideration of body image issues in females and males would be appropriate.

The selection of measures was informed by current understandings of visual imagery and visual WM. They included a self-report measure of the vividness of visual imagery (the VVIQ), computerized versions of the Corsi Block task (Corsi, 1972; Milner, 1971) and the Visual Patterns Task (VPT: Della Sala et al., 1997). We also included a measure of global precedence, and a standard ink-naming colour word version of the Stroop task.

The VVIQ allows participants to self-report their use of visual imagery. There is evidence that it does index visual working memory, but it clearly also implicates elements of long-term memory (Baddeley & Andrade, 2000). Imagery tasks also tend to implicate executive resources (Logie, 1995; Logie, 2003; Pearson, Logie & Green, 1996; Rudkin, Pearson & Logie, 2007; Quinn & Ralston, 1986). Consequently, in order to incorporate more specific measures of visuospatial working memory processing, two direct measures of visual and spatial memory span were used. There is evidence (Della Sala et al., 1999) that the VPT and Corsi tasks measure qualitatively different visual and spatial sub-processes of working memory, and hence together they provide the opportunity to understand relationships with BD in a more sophisticated way. Global precedence is a calculated score reflecting the degree to which a person tends to view visual arrays in a global or a local manner. Global processors may remember faces better (Darling et al., 2009) and process information about faces in a more configural, holistic fashion (Martin & Macrae, 2010). It is included here to see if this preference for broad configural information over small-scale local detail is related to the tendency to misrepresent one’s own body. Somewhat relatedly, Feusner et al. (2010) observed a predisposition to local processing on a face inversion task amongst a sample of individuals with Body Dysmorphic Disorder (BDD).

In addition to direct assessments of visual memory, we also included a measure of resistance to inhibition by irrelevant information—the classic Stroop task (Stroop, 1935). This task was included as an estimate of executive functioning, in particular indexing attention and inhibition (for a review see MacLeod, 1991), two of the so-called executive functions (e.g., Baddeley, 1996; Miyake et al., 2000). Although we acknowledge that executive function is a concept encompassing a diversity of elements, the Stroop task provides a simple and straightforward method of assessing at least some of these components. The classic colour-word interference version of the Stroop task used in this experiment measures the interfering effect of a colour word on the reporting of the colour of ink in which word is presented. Hence the word ‘RED’ might be printed in blue ink, and the participant would be required to report the ink colour as ‘blue.’ Successful completion of the task requires the participant to inhibit the prepotent response (reading out the colour word ‘red’). This task, therefore, does not tap inhibition or orientation to particular concepts, but indexes general inhibitory functioning. Although poor inhibition control has been implicated in eating disorders and obesity, as discussed above, and although the ability to suppress undesired thoughts may be useful in mitigating against body dissatisfaction itself, there is comparatively little evidence linking body dissatisfaction to Stroop performance. Hence it is an open question whether Stroop performance will relate to body dissatisfaction.

In summary, this report presents a study in which we sought to assess the relative importance of various visual imagery, visual memory and executive cognitive factors, with no direct link to eating behavior, in predicting BD in a sample of females. Age and obesity, assessed using Body Mass Index (BMI),^1^1BMI = height/(mass)^2^. were also assessed as predictors, so that an assessment of the role of cognitive factors could be made independent of age and BMI. To do this we adopted two broadly accepted measures of body dissatisfaction: a verbal questionnaire (a short form of the Body Shape Questionnaire, the BSQ-16A: Evans & Dolan, 1993) and a comparison of ideal and current visual-analogue body shape scales.

## Method

### Participants

The sample comprised 111 female participants who were either students or staff of Queen Margaret University, or members of the broader Edinburgh community. Participants took part either voluntarily or in return for course credit. Self-reported colour blindness was an exclusion criterion, and participants were required to have normal or corrected-to-normal vision. Mean age of participants was 26.5 years (SD = 10.2). All participants expressed informed consent in writing, and the research was approved at the Psychology and Sociology Subject area level by the Queen Margaret University Research Ethics Panel.

### Instruments

#### Body shape questionnaire

The 16-item Body Shape Questionnaire (BSQ-16A: Evans & Dolan, 1993) is a self-report scale where participants rate the frequency of various body shape-related feelings over a period of four weeks (e.g., ‘Has worry about your shape made you diet?’). It is a shortened form of the original BSQ-34 (Cooper et al., 1987), which has good psychometric properties (Evans & Dolan, 1993). Ratings are expressed as a 6-point Likert scale ranging from 1 (Never) to 6 (Always), and the reported score is the total of these ratings; thus, BSQ-16A scores have a potential range from 16 to 96.

#### Visual analogue body dissatisfaction (VABD)

This task entailed participants identifying their current and ideal body shape on visual analogue scales (based on Stunkard, Sørensen & Schulsinger (1983)). Dissatisfaction is assessed by subtraction of the ideal from the current rating, quantifying the discrepancy between current and ideal body representation. This method is regarded as an effective measure of BD (e.g., Bulik et al., 2001; Mciza et al., 2005) and correlates with BSQ (Mciza et al., 2005). A higher value indicates a greater degree of dissatisfaction, with the current status being regarded as larger than the desired.

#### Vividness of visual imagery questionnaire

An unaltered version of Marks’ (1973) Vividness of Visual Imagery Questionnaire (VVIQ) was used. Participants are asked to remember 4 scenarios (e.g., ‘think of some relative or friend whom you frequently see … and consider carefully the picture that comes before your mind’s eye’) and for each of these they are asked to attend to 4 key visual features (e.g., ‘The precise carriage, length of step, etc. in walking’). For each of these 16 features, participants give a rating of the intensity of the visual image, on a scale from 1 (‘perfectly clear and as vivid as normal vision’ to 5 (‘no image at all—you only ‘know’ that you are thinking of an object’). Participants carry out the task twice, first with eyes open, and then with eyes closed. The VVIQ score is simply the total score across all 32 items: note that the scoring is counterintuitive; a lower score on the VVIQ represents more vivid imagery.

#### Visual patterns task

The Visual Patterns Task (VPT—Della Sala et al., 1997; Della Sala et al., 1999) is a task designed to assess memory for static visual patterns without the need for participants to recall sequential information. It is a span task, in which the task difficulty is gradually increased until participants fail to recall a pattern. The current study did not use the original VPT, instead an electronic implementation of the VPT was presented.

#### Corsi blocks task

The Corsi Blocks task (CBT—Corsi, 1972; Milner, 1971) requires participants to recall a sequence of locations tapped out sequentially on a board with a number of blocks randomly distributed across its surface, and is considered to be a task that indexes spatial sequential memory as distinct from visual pattern memory (Della Sala et al., 1999). In this study a computerized version of the task was used.

#### Global precedence

Global Precedence was measured by adopting a shortened version of the global/local processing task adopted by Darling et al. (2009) and based on the work of Navon (1977). It provides an assessment of the degree to which irrelevant global information is automatically processed compared to irrelevant local information, controlling for general distractibility; the higher the outcome, the more ‘global’ people are in their outlook, at least to the extent of processing irrelevant global information.

#### Stroop interference

This test was closely modeled after the ink-colour naming word interference task developed by Stroop (1935), and is described in detail in the procedure section. Naming time in a non-conflict condition was subtracted from naming time in a conflicting condition (where ink colour conflicted with the printed word) to give a Stroop Interference (SI) score. A lower score therefore reflects greater ability to inhibit irrelevant information.

### Procedure

After providing informed consent, participants had their BMI measured, and then completed the VABD task: they were presented with a page containing two sets of 9 silhouettes of female figures ranging from severely underweight (1) to severely overweight (9) and asked to indicate which figure most closely matched what they currently looked like on the upper set of silhouettes. They then marked, on the lower set, which item represented the shape that they would most like to look like. After this they completed the paper-based BSQ and VVIQ questionnaires. Participants then took part in the Stroop interference task, in which they were shown a sheet containing a 4 × 6 grid comprising the letter string ‘XXXXX’ represented in either blue, green, red, purple, yellow, or orange ink. Participants were asked to name all of the colours and were timed from start to finish to assess the speed with which they could correctly name all 24. This represented the non-conflicting condition. They were then asked to complete a similar task again, except in this second task, the ‘XXXXXX’ strings were replaced by colour names which conflicted with the ink colour that was to be reported (i.e., the word ‘ORANGE’ was depicted in blue ink).

The remaining three tasks were presented on a a standard laptop computer with a 15.7” display (1,024 × 768 pixels/319 × 239 mm). First, Global precedence was assessed using a task intended to assess participants’ background inclination to process information on a global or a local basis. Participants saw 72 letter displays, in each of which a large letter was comprised of a number of smaller letters (e.g., a letter ‘P’ was formed from a number of individual letter ‘U’s). These displays were modeled on the stimuli used by Navon (1977). Participants were asked to respond by pressing letter keys on the keypad. On 36 trials, the letter display was preceded by an instruction to report the large letter; on the remaining 36 trials participants were told to report the small letter. The instruction used was randomized across trials. For global trials, a local-to-global conflict score was derived by subtracting mean RTs for correct responses for non-conflicting stimuli (where the global and local letters were the same) from correct RTs for conflicting stimuli, yielding a value which increased with the degree to which conflicting local information impaired global processing. For local trials, a global-to-local score was derived in a similar manner reflecting the degree to which conflicting global information impaired local processing. Finally, the global precedence (GP) score was calculated by subtracting the local-to-global conflict score from the global-to-local score. This method yielded a global precedence score which took an increasingly positive value the greater the degree to which global-to-local interference exceeded local-to-global interference.

Following the Global Precedence task, participants undertook the Corsi task. This was implemented as a span task, with difficulty increasing until the point that participants were no longer able to successfully reproduce the spatial pattern. Participants saw a set of 9 black outlines of squares (of side 60 pixels/19 mm) distributed randomly across a white square in the middle of the screen (side: 600/190 mm pixels). A sequence was then traced by randomly highlighting one of these squares at a time by filling it in a green colour for 500 ms. In the first trial, only one item was highlighted. If (on this or any subsequent trial) participants recalled the sequence correctly, the sequence length increased by one item. If an error was made, then a further trial of the same difficulty was presented. Successful recall of this trial enabled the participant to continue at the next level of difficulty, but the procedure stopped after two trials at any difficulty level that were erroneously responded to. Participants were allocated a score representing the maximum sequence length that had been correctly remembered during the task; hence a higher score represents a higher capacity for remembering spatial sequences.

Finally the visual patterns task was undertaken. Participants were presented with grid arrays comprising a number of individual blank squares of side 60 pixels (19 mm). Arrays ranged in size from 2 × 1 cells to a maximum of 6 × 5. In each array, 50% of the squares (at random) were coloured blue. Arrays were visible for a total duration of 250 ms per visible square (filled or unfilled), meaning larger displays were on screen for longer. Following presentation, a blank grid was shown on the screen. Participants were asked to use a standard mouse to select the squares that had previously been shown in blue. Clicked squares changed colour to indicate that they had been selected. Note that use of the mouse for collecting responses in the visual pattern and Corsi tasks could possibly enlist visuo-spatial skills not engaged in the original versions of these measures.

## Results

One participant was initially excluded for providing grossly outlying values (a Stroop interference score of 193, some 49 standard deviations above the mean of other participants). Two further participants did not contribute data to the global/local precedence task due to electronic data collection failures in that task. Table 1 details summary statistics of the principal body image and visual imagery measures in the study for all included participants. Correlations between the variables are reported in Table 2. Global precedence showed no correlations with any of the variables except a small relationship with age (i.e., increased local focus with age). Focusing on relationships between the cognitive measures and measures related to body dissatisfaction, VVIQ correlated positively with BSQ and VABD, showing that higher scores (lower imagery) was related to higher body dissatisfaction. VPT correlated negatively with these measures, showing the same pattern—higher scores (higher visual memory performance) related to lower body dissatisfaction, though effect sizes were smaller than for VVIQ correlations. Correlations between Corsi performance and body dissatisfaction scores were not significant. Stroop interference correlated with both BSQ and VABD. There were also notably strong relationships between VABD, Stroop and BMI.

**Table 1:** Summary statistics of principal variables.

Variable	Valid *N* observations	Mean	SD	Min	Max	Skewness
BMI	110	24.25	4.53	16.85	39.76	1.42
Age	110	26.53	10.20	18	62	1.63
BSQ	110	45.18	17.90	16	89	0.32
VABD	110	1.34	1.23	−1.00	6.00	0.99
VVIQ	110	75.86	20.86	42.00	145.00	0.49
VPT	110	9.93	2.85	4	15	−0.22
Corsi	110	6.26	1.34	4	9	−0.24
GP	108	−65.91	267.73	−930	758	−0.54
SI	110	6.12	3.79	−0.75	19.50	0.95

**Notes.**

BMI Body Mass Index BSQ Body Shape Questionnaire score VABD Visual-Analogue Body Dissatisfaction difference score VVIQ Vividness of Visual Imagery Questionnaire score VPT computerized Visual Patterns Task span Corsi computerized Corsi blocks taskspan GP computerized Global Precedence score SI Stroop interference score

**Table 2:** Correlations between variables. This table shows the Pearson correlation coefficients between the different variables measured in the sample.

	Age	BSQ	VABD	VVIQ	VPT	Corsi	GP	SI
*N*	*108*	*108*	*108*	*108*	*108*	*108*	*106*	*108*
BMI	.33^***^	.03	.53^***^	.12	−.07	−.07	−.06	.36^***^
Age		−.16	.15	.03	−.11	−.22^*^	−.20^*^	.45^***^
BSQ			.56^***^	.41^***^	−.22^*^	−.03	.06	.23^*^
VABD				.34^***^	−.31^**^	−.14	.02	.42^***^
VVIQ					−.11	.03	−.11	.28^**^
VPT						.50^***^	−.01	−.32^**^
Corsi							.09	−.24^*^
GP								.06

**Notes.**

BMI Body Mass Index BSQ Body Shape Questionnaire score VABD Visual-Analogue Body Dissatisfaction difference score VVIQ Vividness of Visual Imagery Questionnaire score VPT computerized Visual Patterns Task span Corsi computerized Corsi blocks taskspan GP computerized Global Precedence score SI Stroop interference score

* *p* < .05.

** *p* < .01.

*** *p* < .001.

To address the fundamental hypotheses of this paper of links between body dissatisfaction and visuospatial working memory, the relative importance of VPT span, Corsi span, global precedence, VVIQ and Stroop interference as predictors of body image was assessed in separate regression analyses upon BSQ and VABD. Age and BMI were included in these analyses as potential covariates of body image. Table 3 reports the results of these analyses. BMI and age were directly entered into the models at Step 1; following this, the remaining cognitive predictors (VVIQ, VPT, Corsi, GP and SI) were allowed to enter using a stepwise procedure (probability criteria for entry/removal were *p* < = .05/ > = .10). To avoid undue influences of individual data points on the regression model, participants with Cook’s D values exceeding 0.037 (i.e., 4/*n*) were excluded from each analysis. The resulting models are detailed in Table 3. The models showed that BSQ scores were reliably (*F*(5, 93) = 12.113, *p* < 0.001: *R*^2^ = .39) predicted by a regression model including VVIQ (*t*(97) = 5.10, *p* < .001), age (*t*(97) = −3.22, *p* = .002), Stroop interference (*t*(97) = 2.42, *p* = .02) and VPT (*t*(97) = −2.04, *p* = .04), and that VABD scores were reliably (*F*(4, 102) = 14.23, *p* < 0.001: *R*^2^ = .37) predicted by a regression model including BMI (*t*(101) = 4.73, *p* < .001), VVIQ (*t*(101) = 3.95, *p* < .001) and VPT (*t*(101) = −3.321, *p* = .001).

**Table 3:** Summaries of the regression models predicting body dissatisfaction. This table summarizes the models generated in the regression procedures.Two regression models are reported, one predicting scores on the body shape questionnaire and one predicting scores on the visual analogue body dissatisfaction measure. Both analyses employed a two-step method, with age and BMI being entered initially as covariates and then the other measures entering in a stepwise procedure. The bottom-most model in each analysis in the table is the final model obtained.

Predicted outcome	Model	Predictor	*b*	*SE b*	*β*	Independent contribution to *R*^2+^
BSQ	1	(Constant)	39.25	9.76		
*Analysis* *N* = 99	Δ*R*^2^ = .04	BMI	.59	.43	.15	.01
		Age	−.38	.20	−.20	.03
	2	(Constant)	12.67	9.56		
	Δ*R*^2^ = .25^***^	BMI	.39	.37	.10	.01
		Age	−.34	.18	−.18	.03
		VVIQ	.40	.07	.51^***^	.25
	3	(Constant)	18.18	9.25		
	Δ*R*^2^ = .07^**^	BMI	.22	.35	.06	<.01
		Age	−.57	.18	−.30^**^	.07
		VVIQ	.34	.07	.43^***^	.18
		SI	1.51	.46	.31^**^	.07
	4	(Constant)	30.38	10.88		
	Δ*R*^2^ =.03^*^	BMI	.25	.35	.06	<.01
		Age	−.57	.18	−.30^**^	.068
		VVIQ	.34	.07	.43^***^	.17
		SI	1.17	.48	.24^*^	.04
		VPT	−1.05	.51	−.18^*^	.03
VABD	1	(Constant)	−1.21	.52		
*Analysis* *N* = *103*	Δ*R*^2^ =.18^***^	BMI	.10	.02	.44^***^	.17
		Age	.00	.01	−.03	<.01
	2	(Constant)	−2.21	.55		
	Δ*R*^2^ =.11^***^	BMI	.09	.02	.40^***^	.14
		Age	.00	.01	−.03	<.01
		VVIQ	.02	.00	.34^***^	.11
	3	(Constant)	−1.16	.61		
	Δ*R*^2^ =.07^**^	BMI	.09	.02	.40^***^	.15
		Age	.00	.01	−.05	<.01
		VVIQ	.01	.00	.32^***^	.10
		VPT	−.09	.01	−.27^**^	.11

**Notes.**

BMI Body Mass Index BSQ Body Shape Questionnaire score VABD Visual-Analogue Body Dissatisfaction difference score VVIQ Vividness of Visual Imagery Questionnaire score VPT computerized Visual Patterns Task span Corsi computerized Corsi blocks taskspan GP computerized Global Precedence score SI Stroop interference score Δ*R*^2^ change in *R*^2^ lowercase *b* regression coefficient *β* standardised regression coefficient

Analysis *N* varies due to exclusion of participants based on Cook’s distance.

+ derived from semipartial correlation. Sample *N* = 108.

* *p* < .05.

** *p* < .01.

*** *p* < .001.

## Discussion

The principal finding of this study is of reliable relationships between some (but not all) visuospatial and cognitive measures and body dissatisfaction. The relationships identified are between body dissatisfaction and respectively visual mental imagery (vivid imagery is associated with lower dissatisfaction) and visual short-term memory (better memory is associated with lower dissatisfaction). In stark contrast to these patterns, there was no evidence of any link between BD and either spatial-sequential short-term memory or global precedence.

These data replicate and considerably extend the results reported by Auchus, Kose & Allen (1993), who described a similar pattern based on an extreme groups analysis whereby the subset of participants who had the most distorted body image representation reported less rich imagery on the VVIQ, but that these groups did not differ in terms of their visual recall. Unfortunately, the robustness of Auchus et al.’s data is unclear, due both to the low sample size in the extreme groups analyses, and the fact that when an inclusive analysis of the data was carried out there were no differences between body image distorters on either VVIQ or visual memory. The current results therefore substantively strengthen our understanding of the role of both visual imagery and memory in body imagery, firstly by convincingly replicating (with a larger sample and more appropriate analysis, therefore obtaining more satisfactory statistical evidence) the link between self-reported visual imagery (VVIQ) and BD, and secondly by establishing a link between short-term visual retention (VPT) and BD; this latter outcome is entirely novel. In doing so, the role of visual cognitive factors in body dissatisfaction is made explicit: participants with better visual memory and greater self-reported mental imagery are less likely to report being dissatisfied with their appearance. Given these results, it is necessary to ask what the mechanisms may be by which mental imagery and visual memory might influence body image: we address each concept separately.

Visual mental imagery reflects the conscious internal representation of visual scenes. Consequently, it is a very difficult phenomenon to assess. Indeed there has been a historic debate between researchers arguing around its very existence as a separate and characteristic entity. One view holds that imagery is entirely verbal and propositional (Pylyshyn, 1981) and hence functions similarly to linguistic propositional knowledge. An alternative view contends that images depict analogue visual scenes in a direct manner (Kosslyn, 1981), based on pictorial representations. Nonetheless, there is some consensus that the VVIQ indexes a reasonably valid construct (McKelvie, 1995), and in doing so provides subjective evidence in favour of pictorial representations. Why might such a system be related to body dissatisfaction? The answer to such a question awaits future research but one possibility is that an efficient pictorial representational system may serve as a mechanism for storing a consistent and reliable visual self-percept that would be robust against biases imposed from the outside world, for example social and media pressure. In contrast, individuals who have less vivid imagery may find their internal representations can be deflected more easily by external pressure and this can lead to greater inconsistency in comparing self-representation with idealized representations. This interpretation is consistent with the claim made by Smeets et al. (2009) that high BD may be associated with less accurate visual imagery for a person’s body.

Our findings can be related to a growing body of literature that demonstrates an important functional role for mental imagery in clinically-related disorders (see Pearson et al., 2013). For example, patients with schizophrenia and high-schizotypy controls show significantly greater vividness of imagery than low-schizotypy controls (Oertel et al., 2009). Participants with depression have also been found to be significantly slower at generating visual mental imagery than non-depressed controls (Cocude, Charlot & Denis, 1997). Furthermore, selective dual-task interference procedures derived from the Working Memory model (Baddeley, 2000; Baddeley, Allen & Hitch, 2011) have been applied to disrupt or reduce clinically-related mental imagery. Dynamic visual noise is a passive interference procedure known to selectively disrupt visual working memory (McConnell & Quinn, 2000; Quinn & McConnell, 2006), and Kemps & Tiggemann (2013) have recently shown that exposure to a hand-held version can significantly reduce naturally occurring food cravings linked to mental imagery. Performance of the demanding visuo-spatial game ‘Tetris’ has also been shown to significantly reduce the occurrence of intrusive mental images for traumatic film material (Holmes et al., 2009).

Some caveats of the current study should be considered. One problem with assessing visual cognition using imagery questionnaires is that the task may not be particularly pure: first, reports are subjective; it is not clear to what extent the self reports are a valid reflection of internal imagery (though it is to be noted that there is evidence that VVIQ scores are affected by manipulations that are known to impact on visuo-spatial working memory (Andrade, Kavanagh & Baddeley, 1997; Baddeley & Andrade, 2000). However, Pearson, Rademaker & Tong (2011) have demonstrated participants can possess a good metacognitive understanding of their own imagery experience, with subjective ratings of image vividness being highly predictive of subsequent impact of imagery on conscious perceptual experience. Brain imaging studies have also linked differences in the introspective experience of mental imagery to differential cortical activation (Cui et al., 2007; Slotnick, Thompson & Kosslyn, 2012). Second, imagery tasks are known to recruit a diverse array of cognitive components including executive functions (Logie, 1995; Logie, 2003; Pearson, Logie & Green, 1996; Rudkin, Pearson & Logie, 2007). Consequently it is possible that the relationship of VVIQ performance and BD is due to some executive factor unrelated to the visual nature of the imagery concerned. The novel finding of an independent, consistent and statistically reliable relationship between VPT performance and BD clarifies the issue: a purer measure of visual memory is reliably related to body dissatisfaction; again, this pattern is consistent with the idea that strong visual representative abilities can protect against body dissatisfaction.

Although superficially it may seem that VVIQ and VPT may index similar functions—one would expect strong visual representational abilities to correlate with vividness of visual imagery—in fact the correlation in our sample between VPT and VVIQ is small and non-significant (*r* (*n* = 110) = −.11, *p* = .254). Bull, Pearson & Hamilton (2007) assessed correlations between VPT and VVIQ as part of a larger-scale study, and reported a slightly higher correlation between VPT and VVIQ (*r* = .22, *p* < .05); they also noted in exploratory factor analysis that VVIQ loaded more on a measure of general executive functioning whilst VPT loaded on a measure of visual representation. On these bases, it seems reasonable to speculate that differences in the profiles of body dissatisfaction relationships between VVIQ and VPT may well centre on the fact that VVIQ has a more substantive executive component, broadly consistent with our findings that VPT appears to be a stronger predictor of VABD (a visual task) than it was the questionnaire-based BSQ.

Stroop interference correlated with every variable in the study apart from global precedence, highlighting its role as a measure of a general inhibitory mechanism. Some of these relationships were very high. The strong and sizeable correlation with age is of little surprise (Comalli, Wapner & Werner, 1962). A strong relationship with BMI is also consistent with previous literature (e.g., Fagundo et al., 2012). In the context of the regression models, Stroop was a moderate predictor of BSQ but was not a significant predictor of VABD. Looking at the high correlations between Stroop interference, BMI, VABD and BSQ, it is plausible to explain this by assuming that the relationship between Stroop interference and body dissatisfaction is heavily driven by the links between obesity and inhibition; VABD is more heavily influenced by actual obesity than BSQ (given that the participant has to select a representation of their current body state) and hence the relationship to Stroop may have been substantially reduced in the regression models because they controlled explicitly for BMI, and more attenuated in VABD prediction rather than BSQ prediction. Given this, it seems reasonable to hypothesize separable relationships between visual short-term memory and body dissatisfaction and between executive functions and body dissatisfaction, a testable hypothesis that should be taken up in future research. One obvious possible explanation is that increased inhibitory control may enable people to inhibit unwanted negative body-related thoughts, thus mediating dissatisfaction.

It is necessary to briefly consider those functions that were not apparently related to either VABD or BSQ, with due deference to the axiomatic absence of evidence not representing evidence of absence. We failed to observe any relationship between spatial sequence memory and BD, nor between global and local processing and BD in our sample. Hence, whilst acknowledging that these are null findings, there is no evidence that the ‘normative discontent’ (Rodin, Silberstein & Striegel-Moore, 1984) that is seen in the general population is related to intrinsic biases in processing orientation, or to individual variations in spatial sequence memory. As far as we are aware this is the first study attempting to link BD with spatial memory and global precedence.

This study restricted its focus to female participants. Hence we must add the caveat that these data do not necessarily generalize to males, and that research on the relationship between visual cognition and BD in males would be warranted in future. Of particular interest here may be the relatively complex patterns of gender difference in visual and spatial memory ability (e.g., Voyer et al., 2007; Cattaneo, Postma & Vecchi, 2006), and it would be worthwhile to tease out relationships between BD, gender and visual cognition in future. In the light of these gender differences, it must be made explicit here that the current data do not support the argument that females are more prone to BD or eating disorders as a consequence of gender differences in visual processing. Such a claim would be groundless, and go way beyond our current claim which is to say that we have observed evidence consistent with a role for visual processing in establishing, maintaining or protecting against BD.

The Working Memory model (Baddeley, 2000; Baddeley, Allen & Hitch, 2011) was advanced earlier as a potentially useful framework for understanding the role of visual imagery in relation to BD. Given the current data, it seems likely that there is a substantive relationship between two components of working memory and BD. Efficient functioning in both the visuo-spatial sketch pad (VSSP) *and* the central executive (CE) seems to be associated with lower BD. The executive processing relationship was stronger for BSQ than for VABD, leading to the tentative conclusion that the BSQ, perhaps by virtue of its use of a verbal questionnaire format, may have more executive demands. Nonetheless, an independent and reliable relationship between Stroop interference to the BSQ demonstrates a relationship between CE and BD that extends beyond the nature of the stimulus materials and implicates the CE in BD. Meanwhile, a relatively small but significant independent contribution of the VPT in predicting both BSQ and VABD demonstrates the importance of visual STM to sustaining BD.

The present study allows a more detailed understanding of the relationship between the VSSP and BD, because only visual STM predicted BD: spatial STM had no such association. There is a reasonable consensus that the VSSP is likely to be divisible into a primarily visual and a primarily spatial system (e.g., Della Sala et al., 1999; Logie, 1995) though there has been debate about the precise nature of these systems and how they interact (e.g., Logie, 2011; Darling, Della Sala & Logie, 2009). There is also evidence that visual and spatial imagery recruit different cognitive systems (Quinn & Ralston, 1986; McConnell & Quinn, 2000). With these patterns in mind, the present results suggest that the systems associated with BD are visual, rather than spatial, in nature: there was no evidence at all for a link between spatial sequential memory (on the Corsi task) and BD.

Recent models of working memory (Baddeley, 2000; Baddeley, Allen & Hitch, 2011) have incorporated a new component known as the episodic buffer (EB), with a role in combining information held in separate stores: the impact of this structure is less clear, and remains to be probed in future research and theorizing. The inhibition and attentional demands of the Stroop task used in the current research are more related to the Central Executive than they are clearly linked to the episodic buffer. As a hypothetical construct thought to require low levels of attention that can link information simultaneously held in different STM and LTM stores, the EB might well be involved in sustaining distorted body-image related representations; for example when an individual may be thinking about their appearance it may provide access to episode-specific information which could easily be a source of biasing or negatively-valenced information. Given the role of the EB role in holding together (or ‘binding’) information held in parallel information streams in order to carry out particular task demands (e.g., Darling et al., 2012; Allen et al., in press), it may be the case that the efficiency of the EB may, speculatively, influence the robustness of self-image representations. However, this is speculation *in extremis*, and a fuller understanding awaits a fuller understanding of the role of the EB in imagery tasks in general, an enterprise that has not really been pursued at the present time (Pearson, 2006). We should also note that the working memory model is not the only notable model of mental imagery that separates visual and spatial processes; this explanation is also largely consistent with other approaches to imagery (Farah et al., 1988; Kozhevnikov, Kosslyn & Shephard, 2005).

In summary, the results of the current study indicate that some measures of visuospatial cognitive function are predictive of body image measures. In order to understand the processes underlying body dissatisfaction, researchers would therefore be well advised to consider visual imagery and visual memory features, and we argue that the well-established working memory model proposes constructs that may help advance this understanding. It is possible that a vivid and veridical visual imagery system offers a degree of immunity to the various insults to body image that are synonymous with life in the modern world, perhaps by providing a robust parallel representation of the body that is either less or differentially affected by messages in the media and society. How this representation is established and maintained is a question for future research. At present we have identified a role for visual STM and the CE but other aspects of cognition may well be involved. As a final note, we wish to make it clear that we completely acknowledge the massive importance of social and media representations to endemic, normative BD, and would not seek to underplay this. However, the finding that *visual* representation is a crucial mediator of BD is also of key importance, and we hope that this finding can be the basis for development of innovative approaches to BD that may help mitigate the pervasive and debilitating social effects of this strikingly prevalent phenomenon.

## Additional Information and Declarations

### Data Deposition

The following information was supplied regarding the deposition of related data: Institutional QMU ‘eData’ repository at http://edata.qmu.ac.uk/21/.
